# Beneficial Role of Rapamycin in Experimental Autoimmune Myositis

**DOI:** 10.1371/journal.pone.0074450

**Published:** 2013-11-12

**Authors:** Nicolas Prevel, Yves Allenbach, David Klatzmann, Benoit Salomon, Olivier Benveniste

**Affiliations:** 1 UPMC Univ Paris 06, UMR 7211, Immunology-Immunopathology-Immunotherapy (I3); F-75005, Paris, France; 2 CNRS, UMR 7211, Immunology-Immunopathology-Immunotherapy (I3); F-75005, Paris, France; 3 INSERM, UMR_S 959, Immunology-Immunopathology-Immunotherapy (I3); F-75005, Paris, France; 4 AP-HP, Hôpital Pitié-Salpêtrière, Biotherapy (CIC-BTi) and Inflammation-Immunopathology-Biotherapy department (I2B), F-75651,Paris, France; University of Düsseldorf, Germany

## Abstract

**Introduction:**

We developed an experimental autoimmune myositis (EAM) mouse model of polymyositis where we outlined the role of regulatory T (Treg) cells. Rapamycin, this immunosuppressant drug used to prevent rejection in organ transplantation, is known to spare Treg. Our aim was to test the efficacy of rapamycin *in vivo* in this EAM model and to investigate the effects of the drug on different immune cell sub-populations.

**Methods:**

EAM is induced by 3 injections of myosin emulsified in CFA. Mice received rapamycin during 25 days starting one day before myosin immunization (preventive treatment), or during 10 days following the last myosin immunization (curative treatment).

**Results:**

Under preventive or curative treatment, an increase of muscle strength was observed with a parallel decrease of muscle inflammation, both being well correlated (R^2^ = −0.645, p<0.0001). Rapamycin induced a general decrease in muscle of CD4 and CD8 T cells in lymphoid tissues, but spared B cells. Among T cells, the frequency of Treg was increased in rapamycin treated mice in draining lymph nodes (16.9±2.2% vs. 9.3±1.4%, p<0.001), which were mostly activated regulatory T cells (CD62L^low^CD44^high^: 58.1±5.78% vs. 33.1±7%, treated vs. untreated, p<0.001). In rapamycin treated mice, inhibition of proliferation (Ki-67^+^) is more important in effector T cells compared to Tregs cells (p<0.05). Furthermore, during preventive treatment, rapamycin increased the levels of KLF2 transcript in CD44^low^ CD62L^high^ naive T cell and in CD62L^low^ CD44^high^ activated T cell.

**Conclusions:**

Rapamycin showed efficacy both as curative and preventive treatment in our murine model of experimental myositis, in which it induced an increase of muscle strength with a parallel decrease in muscle inflammation. Rapamycin administration was also associated with a decrease in the frequency of effector T cells, an increase in Tregs, and, when administered as preventive treatment, an upregulation of KFL2 in naive and activated T cells.

## Introduction

Idiopathic inflammatory myopathies are a heterogeneous group of different diseases, classified into four main categories: dermatomyositis, polymyositis which frequently overlap other connective tissue diseases, immune-mediated necrotizing myopathy, and sporadic inclusion body myositis [1]–[3]. Polymyositis presents endomysial inflammatory cell infiltrates (rich in CD8^+^ T-cells), surrounding and invading non-necrotic muscle fibres, in parallel with a diffuse histocompatibility class I overexpression [4]. The treatment of polymyositis consists of corticosteroids frequently associated with other immunosuppressive drugs [5]. The obligatory and often severe side effects of these drugs, which have to be taken for several months or years, prompted us to propose alternative treatments, which were tested in experimental animal models.

We previously described the induction of experimental autoimmune myositis (EAM) in mice by immunization with partially-purified myosin [6]. This EAM model mimics closely polymyositis, showing muscle weakness and inflammation, and extensive CD8^+^ T cells and macrophages infiltrates. We further demonstrated that it was possible to induce the disease by adoptive transfer of unsorted lymph node cells or *in vitro* restimulated sorted CD4+ T cells in wild type mice. In this EAM model, we also demonstrated that regulatory T cells (Treg) can ameliorate the disease phenotype [6].

Tregs represent around 10% of CD4^+^ T cells. Tregs in mice and humans express the transcriptional factor FoxP3 encoding for the forkhead/winged-helix transcription factor scurfin [7]–[9]. Quantitative or qualitative deficits in the Treg compartment have been reported in many mouse models of autoimmune diseases and in humans [10]–[14].

Rapamycin is a known potent immunomodulator with less side effects compared to other immunosuppressants (e.g. ciclosporin). Rapamycin has been used for a decade in transplant patients to prevent graft rejection [15]. *In vivo*, rapamycin has been described to increase the percentage of Treg [16]–[18].

The aim of this study was to test the efficacy of rapamycin *in vivo* in our EAM model and to investigate the effects of the drug on different immune cell sub-populations, with the ultimate goal of developing a new therapeutic approach for polymyositis.

## Methods

### Mice

Six to ten-week-old female BALB/c mice were purchased from Janvier Laboratories. All mice were kept under specific pathogen free conditions and manipulated according to European council directive 86/609/EEC. The study was approved by the Regional Ethical Committee 3 of Ile-de-France.

### Evaluation of Muscle Strength

Muscle strength was evaluated using an inverted screen test as described previously [6], [19] one day before mice sacrifice. Briefly, a mouse was placed at the center of a wire mesh screen, which was inverted horizontally and the time to fall off the screen was recorded. All mice were evaluated independently by one investigator who was blinded to the immunization protocol.

### Preparation of Myosin

Myosin was partially purified according to the method previously reported [6]. Briefly, A total of 30 ml of chilled 0.3 M/L KCl–0.15 M/L sodium phosphate buffer (pH 6.5) was added to 10 g of minced muscle tissue and kept on ice for 45 minutes. This homogenate was centrifuged at 15,000 rpm for 30 minutes at 4°C, and the supernatant collected. The filtrate was then diluted with 15 volumes of chilled Milli-Q-filtered (Millipore, France) purified water to precipitate the myosin. The precipitated myosin was collected by centrifugation at 5000 rpm, dissolved in 0.5 M/L KCl, and stored at −80°.

### Mice Immunization Protocol

Mice were immunized 3 times, at 1-week intervals, with 200 µl of phosphate-buffered saline (PBS) containing 1 mg myosin (or with 200 µl of PBS in control groups) emulsified with an equal volume of complete Freund's adjuvant (CFA, Sigma). Myosin was purified from mouse muscle, an extract containing 80% of myosine. These emulsified preparations were injected on bilateral sides of the hind footpads (first immunization), the tail base (second immunization), and flanks (third immunization). Pertussis toxin (Sigma) was injected intra-peritoneally (500 ng in 200 µl of saline) during the first immunization. Ten days after the last immunization, muscle blocks were collected for histology.

### Treatments of mice

In the preventive treatment (Figure S1A), mice received daily oral placebo (200 µl of water only, referred to as controls) by force-feeding with a cannula, 1 mg/kg/day of rapamycin (referred to as Rapa 1 mg), or 3 mg/kg/day of rapamycin (referred to as Rapa 3 mg) diluted in 200 µl of water, from the day before the first myosin immunization to day 10 after the third myosin immunization (date of their sacrifice). In curative treatment (Figure S1B), mice received daily oral rapamycin (3 mg/kg/d) from one day after the third myosin immunization (at a date where all mice presented inflammatory infiltrates within muscle, Figure S2A) to day 10 after the last immunization (date of their sacrifice). Control animals received 200 µl of water daily.

### Histological Grading of Inflammatory Lesions and Immunohistochemistry

For each animal, paraffin sections from three muscles (gastrocnemius, quadriceps, and triceps) were examined histologically for the presence of mononuclear cell infiltrates and necrosis of muscle fibers. For each sample, eight groups of serial sections were cut at a 200 µm distance. From each group, one 5 µm-thick section was used for hematoxylin and eosin staining. All fields of each section were analysed. The histological severity of inflammation in each muscle block was graded as previously described [20] by Matsumoto: Grade 1 = involvement of a single muscle fiber or <5 muscle fibers; grade 2 = a lesion involving 5 to 30 muscle fibers; grade 3 = a lesion involving a muscle fasciculus; and grade 4 = diffuse, extensive lesions. When multiple lesions with the same grade were found in a single muscle block, 0.5 points were added to the grade. All slides were evaluated independently by two investigators who were blinded to the immunization protocol that had been used. When results were discordant, the slides were re-evaluated by two pathologists and a consensual score was given after review. For each muscle analysed, only the highest score was reported. The maximum score is 4.5. Theoretically, the maximum score is 4 (i.e. diffuse, extensive lesions) but when multiple lesions with the same grade were found in a single muscle block, 0.5 points were added to the grade. If the score is different, the slides are discussed during a pathologists meeting and a consensual score is given after review

For immunohistological analyses, serial cryosections were fixed with cold acetone and immunostained with rat anti-mouse CD4 (clone RM4-5, BD Biosciences), rat anti- mouse CD8b (clone 53-5.8, BD Biosciences), rat anti-mouse CD45R/B220 (clone RA3-6B2, BD Biosciences), rat anti- mouse CD11b (clone M1-70.15, BD Biosciences) monoclonal antibodies (BD Biosciences), and rabbit anti-laminin (Sigma) polyclonal antibodies. APC and PE conjugated anti-rat (Ebiosciences) and FITC-conjugated goat anti-rabbit IgG (HL, Jackson ImmunoResearch) were used as secondary antibodies. Nuclei were counterstained with 4,6-diamidino- 2-phenylindole (DAPI) and appeared in blue.

### Flow Cytometric Analysis

The following antibodies were used for flow cytometry analysis: PE-labeled anti-CD4 (RM4-5, BD Biosciences), anti-CD8 (clone 53-6.7 BD Biosciences), anti-CD19 (clone 1D3, BD Biosciences), anti-CD62L (clone MEL-14, BD Biosciences), anti- FoxP3 (FJK16-second, eBioscience), anti-Ki-67 (SolA15, Ebiosciences), FITC-labeled anti-CD4 (clone RM4-5 BD Biosciences), anti-CD44 (clone MEL-14, BD Biosciences), APC-labeled anti-CD25 (clone PC61 BD Biosciences), PercP CD4 (clone RM4-5, BD Biosciences), and biotinylated anti-CD25 (clone 7D4, BD Biosciences) revealed with streptavidin-Cy-Chrome (BD Biosciences) and appropriate isotype antibody controls. At least 100,000 events were acquired on a FACS LSRII (Becton Dickinson) and analyzed using FlowJo software.

### Suppression Assays

Splenocytes harvested from control or rapamycin treated mice, were irradiated (20 Gy) and plated onto 96-well plates (10^5^ cells/well). T effector cells (Teff, lymph node cells harvested from naïve mice) at a constant number (10^5^ cells/well) were then added with a varying number of *in vitro* expanded Treg cells (CD4+ CD25+ sorted cells) to provide Teff/Treg ratios of 1∶0, 1∶1, 2∶1, 5∶1, and 10∶1. Soluble anti-CD3 mAb (145–2C11, PharMingen) at a concentration of 2 µg/ml provided the polyclonal stimulus for proliferation. Cells were incubated in RPMI with 10% fetal bovine serum in a total volume of 200 µl. After 3 days of culture, 1 µCi of 3H-thymidine (Amersham Biosciences) was added for the final 12 hours to assess proliferation.

### Quantitative PCR

Total RNA was extracted from tissues or purified cell populations with TRIzol reagent (Invitrogen) according to the manufacturer's instructions, treated with DNase using a DNA-free kit (Ambion) and subjected to reverse transcription with SuperScript III reverse transcriptase and random hexamers (both from Invitrogen). cDNA was analyzed in duplicate by QPCR amplification using Power SYBR Green PCR Master mix (Applied Biosystems).

Data were analyzed by comparative quantification with MxPro software (Genomics, Agilent Technologies).

### Primer sequences

We looked at the expression of KLF2 in different cell populations using the following primers: forward 5′-AGCCTATCTTGCCGTCCTTT-3′, reverse 5′-CGCCTCGGGTTCATTTC-3′. We normalized our results with the expression of hypoxanthine-guanine phosphoribosyl transferase (HGPT) in the same cells, using the following primers: forward 5′-CTTCCTCCTCAGACCGCTTT-3′, reverse 5′-ACCTGGTTCATCATCGCTAA-3′.

### Statistical Analysis

Data are presented as mean (±SD) for continuous variables and percentage for qualitative variables. Non-parametric Mann–Whitney test was used to compare variables. A p value<0.05 was considered significant. Statistical analyses were performed using GraphPad Prism version 4.0 and Instat version 3.0 for Windows (GraphPad Software, San Diego, CA, USA).

## Results

### Preventive treatment with rapamycin results in a reduction of inflammatory infiltrates parallel to an increase in muscle strength

The first step in our study was to investigate preventive effects of rapamycin on EAM by testing two doses of the drug: 1 and 3 mg/kg/day.

Rapamycin was very well tolerated by all mice for the entire duration of the administration protocol, for instance no weight loss was observed (data not shown).

**Figure 1:**
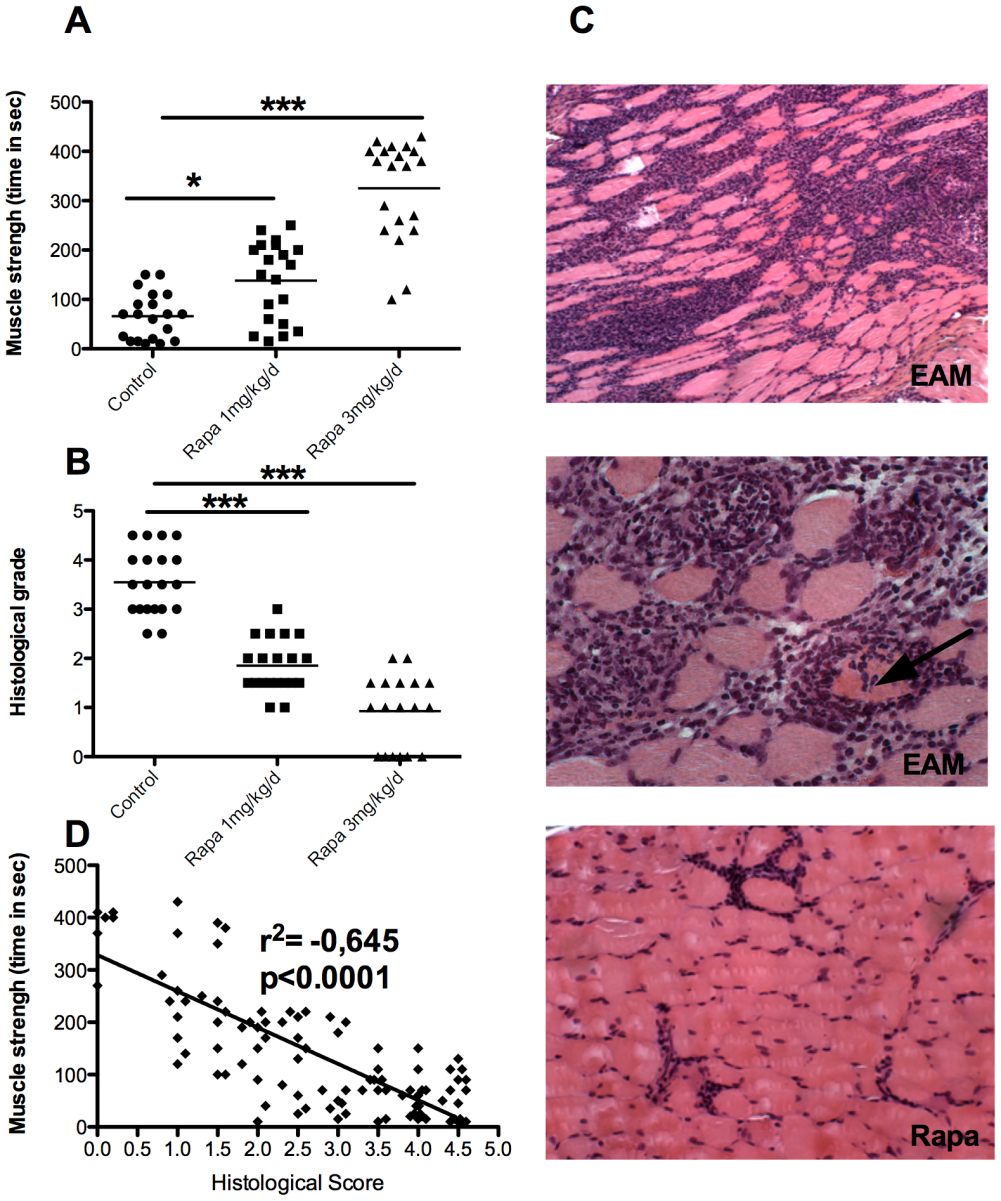
Preventive administration of rapamycin permits to decrease severity of EAM. A: strength (time to fall) improvement in rapamycin-treated mice. B: gastrocnemius, quadriceps, and triceps muscle inflammatory infiltrates evaluated by histological grading after hematoxylin-eosin staining as illustrated in C. C: the first two upper images represent a gastrocnemius section of an EAM mouse untreated with a histological score of 4 (HE, ×20). The middle picture also shows an invaded/tunnelized fiber (arrow, HE, ×40). The third image panel represents a gastrocnemius section of a EAM mouse treated with 3 mg/kg/day of rapamycin, displaying a histological score of 1 (HE, ×20). D: correlation between muscle strength (time to fall) and histological grade of inflammation.

Compared with controls, a dose-dependent increase in muscle strength was observed (time to fall: controls 66±46 seconds, Rapa 1 mg 138±80 seconds p<0.05; Rapa 3 mg 325±1001 seconds; p<0.001 for control vs Rapa 3 mg, Figure 1A). This was accompanied with a parallel decrease in muscle inflammation attested by a lower histological score in EAM animals treated with rapamycin (controls, 3.55±0.67, Rapa 1 mg, 1,85±0.54, p<0.001, Rapa 3 mg, 0.92±0.69; p<0.001 for control vs Rapa 3 mg, Figure 1B and Figure 1C). In the treated and control animals, clinical and pathological scores showed good correlation (R^2^ = −0.645, p<0.0001, Figure 1D).

### Preventive treatment with rapamycin induced a general reduction in frequency of T cells

**Figure 2:**
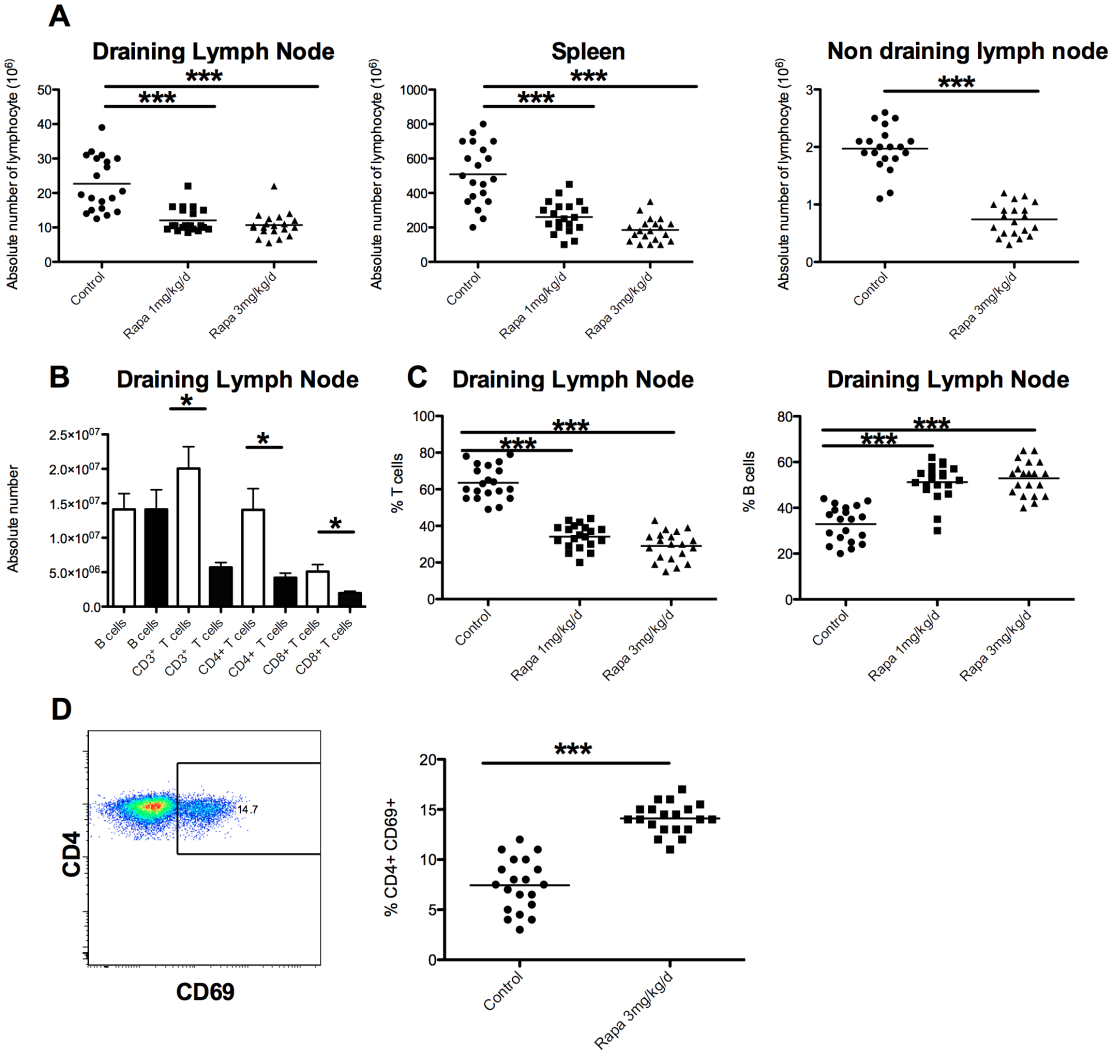
Rapamycin treatment induced a T cells lymphopenia sparing B cells. A: absolute lymphocyte number in draining lymph nodes, spleen, and non-draining lymph nodes is decreased in rapamycin-treated mice compare to controls in a dose-dependent fashion. B: quantification of the different sub-populations of lymphocytes: B lymphocytes (B, CD19^+^B220^+^), T (CD3^+^), T CD4^+^ (CD3^+^CD4^+^) and T CD8^+^ (CD3^+^CD8^+^) cells. * p<0.05, *** p<0.001. White histogram bars: control mice, black histogram bars: rapamycin treated mice (3 mg/kg/day). C: percent of T or B lymphocytes in draining lymph nodes. D: Representative dot plot of flow cytometry analysis of draining lymph nodes for percentage of pre-activated CD4^+^ T cell (CD3^+^CD4^+^FoxP3^−^CD69^+^) and percentage of pre-activated CD4^+^ T cell (CD3^+^CD4^+^FoxP3^−^CD69^+^) in controls and rapamycin (3 mg/kg/day) treated mice.

To explore the mechanisms involved in the improvement of muscle inflammation, we analysed lymphocyte population changes induced by rapamycin in different lymphoid organs. As shown in Figure 2, by comparison to controls, rapamycin induced a significant decrease in the absolute number of lymphocytes in every tested lymphoid organ (draining or non draining lymph nodes and spleen, Figure 2 A) with a dose effect. For instance, in popliteal draining lymph nodes, the number of total lymphocytes was 21.8±6.9×10^6^ in control mice compared to 11.5±2.7×10^6^ (p<0.001) in Rapa 1 mg-treated animals.

The lymphopenia observed in rapamycin-treated mice was due to a decrease in CD4 and CD8 T cell populations, i.e. among CD3^+^T cells, the decrease in number was equally partitioned between CD4^+^ and CD8^+^ T cells (Figure 2B), leading to a significant decrease in the overall T cell frequency (Figure 2C). Rapamycin had no effect on B cells whose absolute number remained stable (Figure 2B) and percent relative to T cells increased (Figure 2B) due to the reduction in T cell frequency. CD4^+^/CD8^+^ T cells ratio was not different in Rapa 1 and 3 mg/kg/day compare to the control group (figure 2B).

In the CD4^+^ T cell compartment, we observed an increase of pre-activated CD4^+^ T (CD69^+^) in popliteal draining lymph nodes in rapamycin treated mice, 14.1±1.5% vs. 7.4±2.6% in controls mice (p<0.001) (figure 2D).

### Despite CD4^+^ T cells decrease, rapamycin spares Tregs

**Figure 3:**
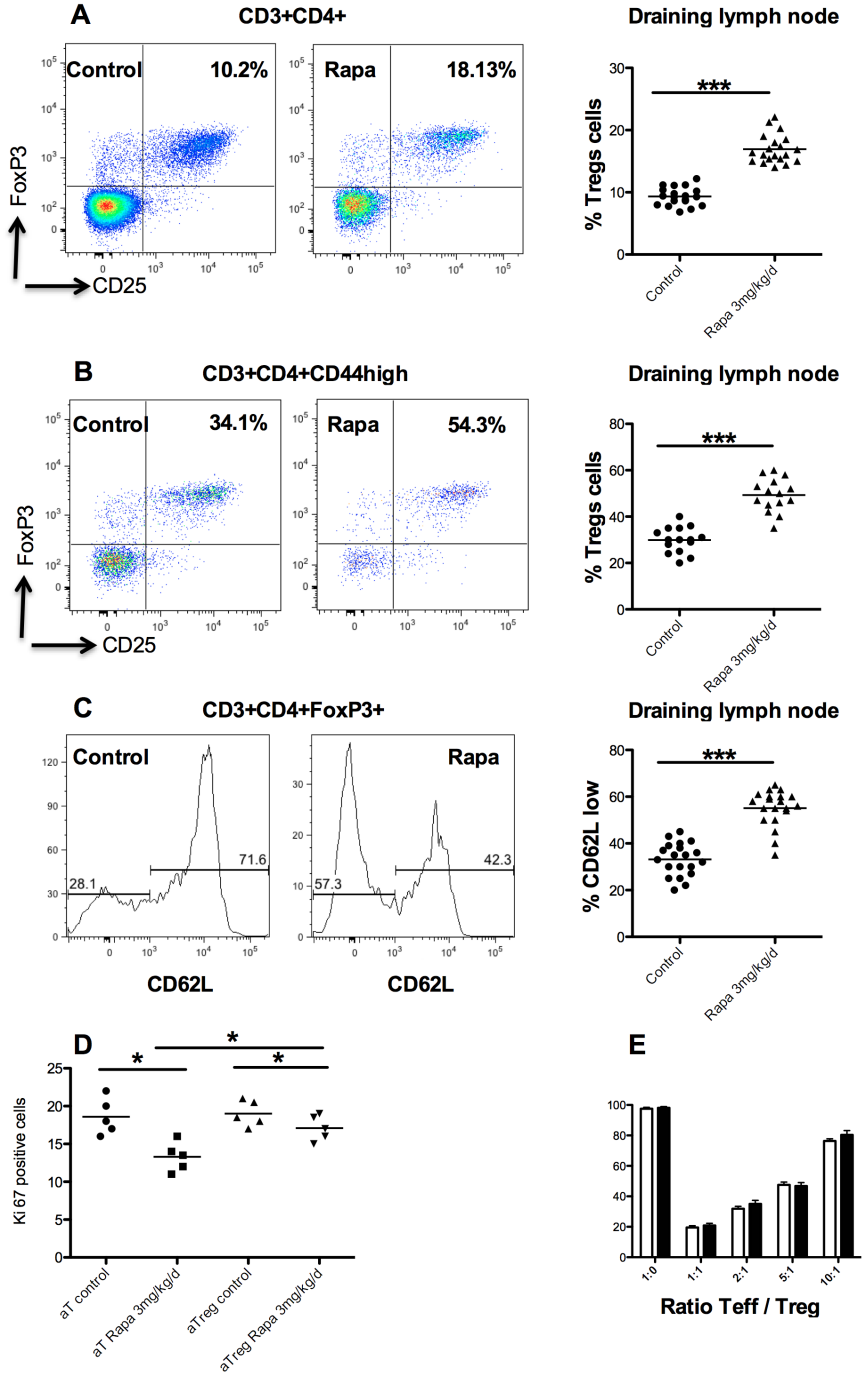
Effect of rapamycin on Treg cells. Representative dot plot of flow cytometry analysis of draining lymph nodes for percent of Treg (CD4^+^CD25^+^FoxP3^+^) in CD4^+^ (A) and in CD4^+^CD44^high^ (B) in controls and rapamycin (3 mg/kg/day) treated mice. Percent of Treg (CD4^+^CD25^+^FoxP3^+^) in CD4^+^ (A, right) and in CD4^+^CD44^high^ (B, right) in controls and rapamycin (3 mg/kg/day) treated mice. C: Histogram representative of the difference in activation of Treg (CD62L^low^ cell) and naive Treg (CD62L^high^) in controls and rapamycin (3 mg/kg/day) treated mice. D: Suppressive test. Horizontal lines indicate means. Suppressive activity of sorted CD4^+^CD25^+^ T cells (Treg) from controls (white bars) and rapamycin (3 mg/kg/day, black bars) mice on the proliferation of autologous CD4^+^CD25^−^ T cells (responders) stimulated with irradiated (15 Gy) splenocytes. Proliferation of responder cells (Teff) was measured by 3H-Tymidine incorporation (counts per minute, cpm). Results are indicated as percentage inhibition (±SD) compared to a max (ratio 1∶0). Different Teff∶Treg ratios were tested. E: Percent of Ki-67positive cells (i.e. proliferative cells) in activated effector (aT) and activated regulatory (aTreg) T cells in controls and rapamycin (3 mg/kg/day) treated mice.

Because changes were more marked in animals treated with rapamycin 3 mg/kg/day (Rapa 3 mg), we focused our efforts in comparing this group to control mice. An increased percent of Treg (defined as CD4^+^CD25^+^FoxP3^+^ T cells) was observed in the CD4^+^T cells compartment in Rapa 3 mg mice compare to controls (16.9±2.2% vs. 9.3±1.4%, p<0.001, Figure 3 A) in draining lymph nodes. In the activated CD4^+^ T cells compartment (CD3^+^CD44^high^), a higher proportion of Treg was observed in rapamycin treated mice (Figure 3B). In the Treg compartment, the percentage of activated Treg (CD62L^low^CD44^high^Treg) was increased in rapamycin treated mice compare to control (58.1±5.78% vs. 33.1±7%, p<0.001, Figure 3C).

As rapamycin increased the relative frequency of Tregs, we tested the suppressive activity of this subset of T cells. To test Treg function, suppressor assays were performed, showing that regulatory functions of Treg were not modified by rapamycin treatment compared to control (37% vs. 39% of suppression at 2∶1 suppressor∶effector ratio, Figure 3D).

**Figure 4:**
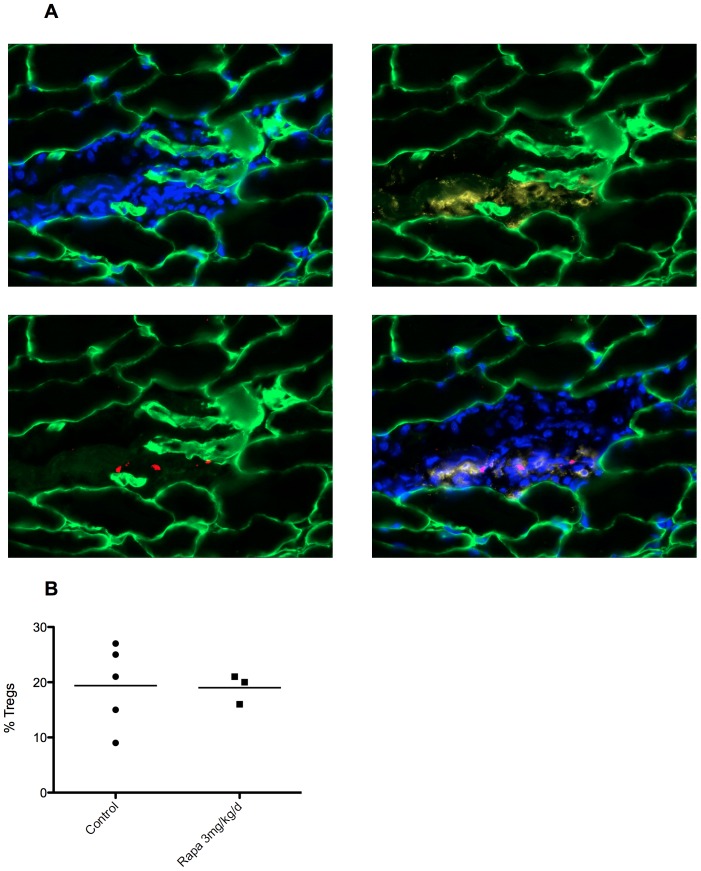
Rapamycin treatment did not alter muscle infiltrates composition. A: Gastrocnemius muscle fiber membranes are stained with an anti-laminin antibody (green). Nuclei were counterstained with DAPI (blue). Cells of muscular inflammatory infiltrates are stained with anti-CD4 (yellow) and with anti-FoxP3 (red) antibodies. B: Percent of Tregs (CD4+FoxP3^+^) in muscle inflammatory infiltrates.

In the activated regulatory T cells (CD4^+^FoxP3^+^CD62L^high^T cells), we observed (Figure 3E) a decrease of Ki-67 cells (19±1.6 vs 17±1.4, p<0.05) in rapamycin treated mice compared to control mice. We observed the same effect in effector T cells (CD4^+^FoxP3^−^CD62L^high^T cells, Figure 3E). Furthermore, inhibition of proliferation under rapamycin is more important in effector cells compared to Tregs cells (p<0.05). Because rapamycin treated mice have an increase percentage of Treg in draining lymph nodes, we wanted then to analyse Treg infiltration within muscles. Infiltrates were mainly composed by macrophages (CD11b), and CD8+ T cells (data not shown). Some CD4^+^ T cells were also observed (figure 4A), but Treg (CD4^+^FoxP3^+^ cells) remained sparse (figure 4A). Like for total CD4+ T cells in draining lymph nodes, there was a decrease in the absolute number of Tregs within the muscle following treatment with rapamycin even if the percentage seemed to remain stable. For 5 different mice in both groups (rapamycin treated and control), one representative cryosection was selected from the most inflamed muscle and FoxP3 and CD4 positive cells were counted. In control mice, on 5 representative sections, we counted 16 FoxP3 positive cells out of 81 CD4 positive cells. Treg represent then 20% of CD4+ cells. In rapamycin treated mice, we counted significantly less CD4 and FoxP3 positive cells since 2 mice had no detectable inflammatory infiltrate and the 3 remaining had 7 FoxP3+ cells on 37 CD4+ cells), but the percentage remained roughly the same, 19% vs 20% in treated vs. control mice (p>0.1 Figure 4B).

### Rapamycin modified the KLF2 pathway

An increase in the percent of Treg but also in the frequency of pre-activated CD69^+^ effector T cells (figure 2D) was observed in draining lymph node of Rapa 3 mg mice whereas muscular infiltrates decreased. Therefore, we hypothesized that a T cell trafficking modification under rapamycin treatment could represent one of the mechanisms of action of the drug.

**Figure 5:**
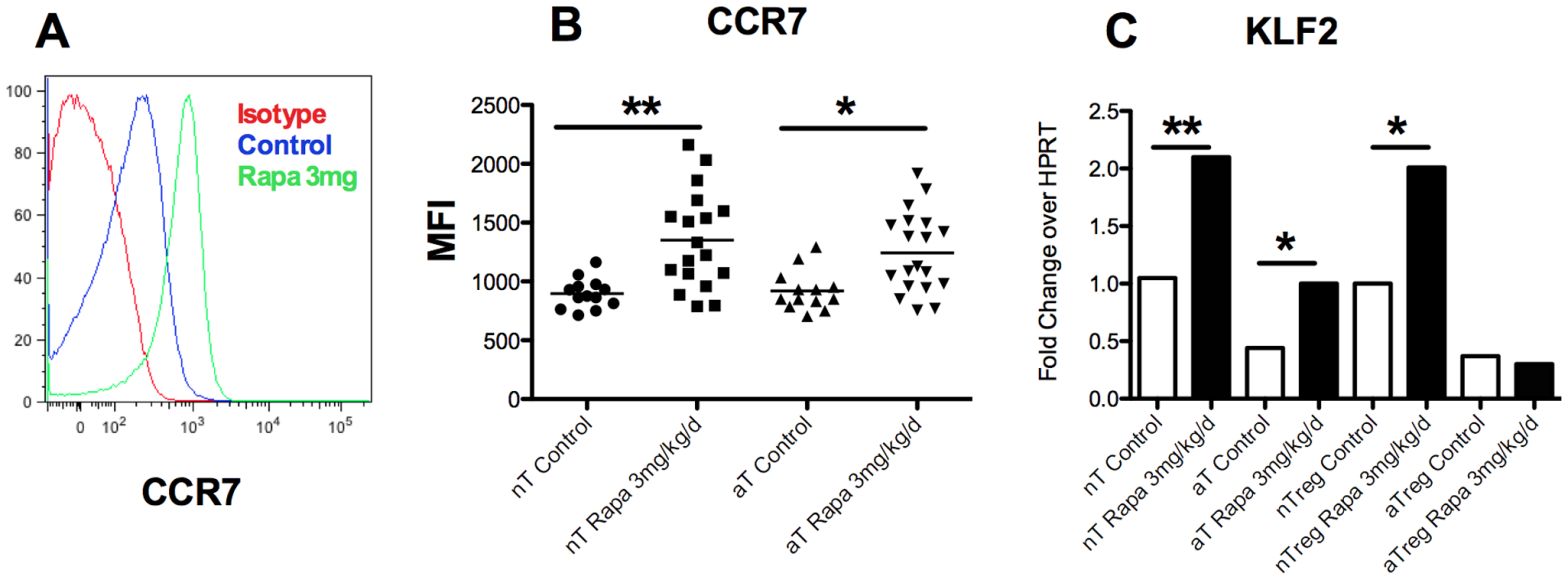
Change in KLF2 pathway induced by rapamycin treatment. A: Histogram plot of the shift of CCR7 expression in controls and rapamycin-treated (3 mg/kg/day) mice showing naive T cell subset (nT:CD62L^high^CD44^low^). B: Shift of CCR7 expression in controls and rapamycin-treated (3 mg/kg/day) mice in naive T cells (nT:CD62L^high^CD44^low^) an activated T cells (eT:CD62L^low^CD44^high^). C: Q-RT-PCR of KLF2 in naive T cells (nT:CD62^high^CD44^low^), activated T cells (aT: CD62^low^ CD44^high^), naive regulatory T cells (nTreg:CD62^high^), activated Treg cells (aTreg: CD62^low^) in controls and rapamycin treated (3 mg/kg/day) mice.

KLF2 is a transcription factor which controls three important genes for lymphocyte homing: the integrins CCR7 and CD62L, involved in the entry of T cells in lymph nodes, and S1P1, involved in their exit [21]. mTOR is known to negatively regulate KLF2^21^. We have then tested if rapamycin is able to modulate these proteins. After 25 days of treatment with rapamycin, an up-regulation of CCR7 was observed at the surface of CD44^low^ CD62L^high^ naive T cell (897±126 mean fluorescent units [mfi] in control mice vs. 1352±414 mfi in rapamycin treated mice in draining lymph nodes, p<0.001) but also in CD62L^low^ CD44^high^ activated T cells (920.2±168 mfi vs. 1243±343 mfi, p<0.05, Figure 5A and B). This result is in line with the observed increase level of KLF2 transcripts (Figure 5C) in CD44^low^ CD62L^high^ naive T cell (1.05±0.09 vs. 2.1±0.14, fold change over HPRT; p<0.05) and in CD62L^low^ CD44^high^ activated T cell (0.44±0.08 vs. 1.02±0.18, fold change over HPRT; p<0.05). In naïve regulatory T cells, we observed an increase of KLF2 (1±0.15 vs 2.05±0.19, p<0.05) but not in activated Tregs (Figure 5C).

### Curative treatment with rapamycin allows for a reduction of inflammatory infiltrates, increase in Treg frequency, and a simultaneous increase in muscle strength

**Figure 6:**
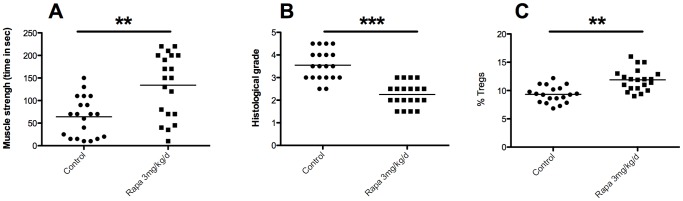
Beneficial effect of rapamycin (3 mg/kg/day) in curative treatment of EAM. Rapamycin (or water for control animals) was given orally for 10 days at a dose of 3 mg/kg. A: strength of mice evaluated by inverted screen test (time to fall in seconds). B: Gastrocnemius muscle inflammatory infiltrates evaluated by histological grading after haematoxylin-eosin staining. C: Percent of Treg cells (CD3^+^CD4^+^CD25^+^FoxP3^+^) in draining lymph nodes from controls and rapamycin-treated (3 mg/kg/day) mice.

The final step of our study was to test the effect a curative treatment in mice with established EAM (i.e. once EAM is induced by myosin immunization). At the third immunization, all mice presented inflammatory infiltrates within muscle (Figure S2A). Compared to control mice, we observed an increase of muscle strength with rapamycin (time to fall: 64±43 seconds vs. 134±70 seconds, p<0.01, Figure 6A) and a decrease of muscle inflammatory infiltrates (3.5±0.6 vs. 2.2±0.5, p<0.001, Figure 6B). However, muscular infiltrate was higher in the curative treated mice than the preventive treated mice (2.2±0.5 vs. 0.92±0.69, p<0.001). In the treated and control animals, clinical and pathological scores showed good correlation (Figure S2B) like during the preventive treatment. As in the preventive treatment, we observed an increase in Treg frequency in draining lymph nodes of rapamycin treated mice (11.9±1.92% of Tregs) compared to control mice (9.33±1.4% of Tregs; p<0.001, Figure 6C). However, curative treatment had no significant impact on the KLF2, CCR7 cell surface expression comparatively to preventive treatment (data not shown). As for the preventive treatment, we observed around 20% of Treg (FoxP3+) among the CD4+ cells within the muscle inflammatory infiltrates in treated and untreated mice (data not shown).

## Discussion

In this study we showed that rapamycin decreases the severity of myositis (both clinical and pathological end points of the disease) in our model of EAM. This was achieved with both preventive and curative treatment. The amelioration of the disease phenotype was associated with a decrease in effector T cells (Teff) which was not compensated by an increase of proliferation of activated cells and a relative increase of Treg cells in draining lymph nodes. In addition, there is a change in the KLF2 pathway in preventive treatment.

A beneficial role of rapamycin in mice models of autoimmune diseases like experimental autoimmune encephalomyelitis (EAE) [17], auto-immune arthritis [22], or NOD mice (diabetic model) [23] have already been reported. Mechanism involved in disease improvement includes a lymphopenia [21] as we observed in our study. Unlike other publication [24], we observed only a modulation of T cells, but not of B cells. Such discrepancy maybe explained by the fact that rapamycin inhibits mainly proliferating cell [6], [21]. Rapamycin also induced an increase in the frequency of Treg cells in draining lymph nodes of EAM mice. Others have reported such observation in human and mice for other diseases but not for EAM and polymyositis [16]–[18], [23].

The reason why anti-proliferative effect of rapamycin spare Treg may be explained by the fact that mTOR pathway is not crucial for Treg proliferation [24], differently from effector T cell. It is confirmed by the greater decrease of Ki-67^+^ (proliferative) cells among Teff cells compared to Tregs. On the other hand, we did not observe an increase percentage of Treg in muscular compartment. This is in contrast with other models, in particular in EAE [17] in which Treg were strongly increased in the central nervous system and to a less extend in draining lymph nodes. Differences in immunisation protocols, in the site of inflammation (muscles vs. central nervous system), and in the treatment regimens, may induce different patterns of T cells proliferation and trafficking [25].

In our study, we also observed an increase in the expression of T cell homing marker KLF2 in rapamycin treated mice (in preventive treatment). KLF2 is a transcription factor which positively regulate the expression of the integrin CD62L and the chemokine receptor CCR7 involved in the entry of lymphocytes in lymph nodes [26]–[29]. Notably KLF2^−/−^ mice have decrease naïve T cell and accumulation of T cells within tissues especially muscles because of an over expression of CCR-3 an CCR-5 [30]. Indeed, recent *in vitro* data showed that the KLF2 is down regulated by mTOR pathway and that rapamycin increased its expression [31]. For the first time, we show *in vivo* an increase of KLF2, expression in naive and effector T cells with the administration of rapamycin.

Thus together our data suggest that rapamycin improved EAM by inhibiting effector T cell responses, including autoreactive T cells proliferation (Ki-67^+^ cells), but also maintaining effector T cells in draining lymph nodes. Furthermore, by sparing Treg proliferation in draining lymph node, rapamycin permits to increase Treg/Teffector cells ratio which is crucial to improve Treg-mediated immunosuppression [24].

Curative treatment also permitted to decrease the severity of the myositis but less efficacious than the preventive treatment did. This discrepancy is probably due to the fact that in curative regimen muscular inflammation is already established whereas by definition in preventive settings treatments avoid muscular infiltrates and/or that the duration of treatment is longer in the preventive protocol.

Rapamycin has been extensively used as immunosuppressant in transplantation. In recent years, a growing interest in the sparing effect of rapamycin on Tregs [32] has promoted the initiation of several clinical trials in which the drug has been tested in autoimmune diseases like diabetes (Rapamune in Type 1 Diabetes, National Institute of Allergy and Infectious Diseases, number NCT00525889). To date, no cases were reported in which rapamycin was used to treat polymyositis, nevertheless, two cases of refractory dermatomyositis treated by rapamycin with good results have been published [33], [34], suggesting that the drug could have a beneficial effect in patient affected by autoimmune myositis.

### Conclusion

Those observations suggest that rapamycin may represent an effective new therapeutic approach in patients with polymyositis, allowing to reduce steroid administration. This approach may be particularly beneficial in this patient population, as a deficiency in Treg frequency has been reported in individuals affected by polymyositis [35].

## Supporting Information

Figure S1 **Description of preventive treatment (A) and curative treatment (B) by rapamycin in the EAM model.**
(TIFF)

Figure S2 **Kinetic of histological score and correlation between muscle strength (time to fall) and histological grade.** **A**: histological score of animals over time after 3 weekly immunizations against myosin at day 0, 7, and 14 (D0, D7, and D14, respectively). B: correlation between muscle strength (time to fall) and histological grade of inflammation after curative treatment of EAM.
(TIFF)
