# Correction: Beneficial Role of Rapamycin in Experimental Autoimmune Myositis

**DOI:** 10.1371/annotation/3eb548ab-c781-4784-9dee-b5a27f7e1643

**Published:** 2014-01-14

**Authors:** Nicolas Prevel, Yves Allenbach, David Klatzmann, Benoit Salomon, Olivier Benveniste

The affiliation for fifth author, Olivier Benveniste, is incorrect. The correct affiliation is AP-HP, Hôpital Pitié-Salpétrière, Service de Médecine Interne 1, Centre de Référence Maladies Neuro-Musculaires, U974, Université Pierre et Marie Curie and Inflammation-Immunopathology-Biotherapy department (I2B),Paris, France

