# Supplementary figures and images for: Beneficial Role of Rapamycin in Experimental Autoimmune Myositis
Source: PLoS One. 2013 Nov 12;8(11):e74450. doi: 10.1371/journal.pone.0074450 (PMC3827074; doi:10.1371/journal.pone.0074450)

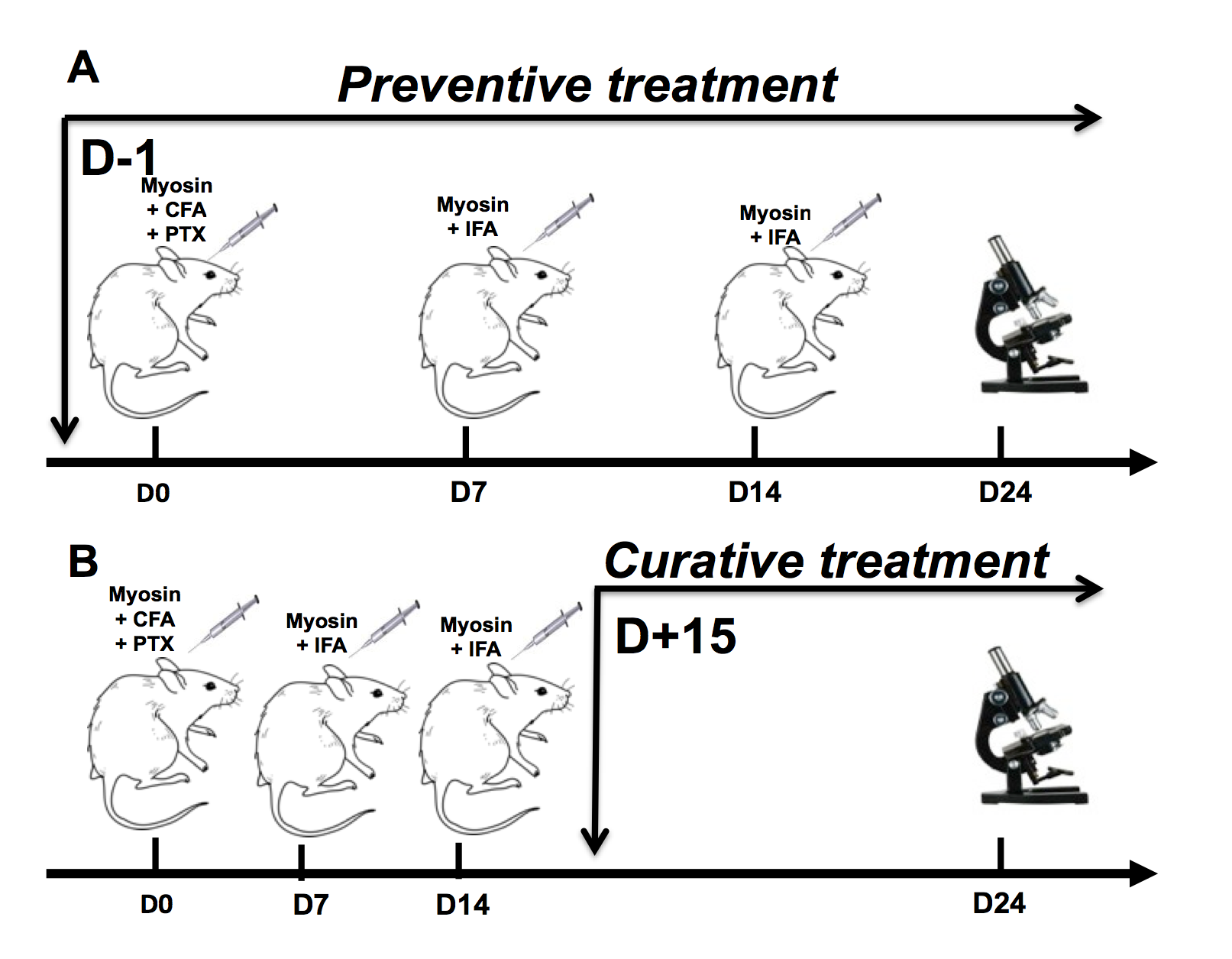

Supplement: Figure S1 — Description of preventive treatment (A) and curative treatment (B) by rapamycin in the EAM model. (TIFF) [file pone.0074450.s001.tif]

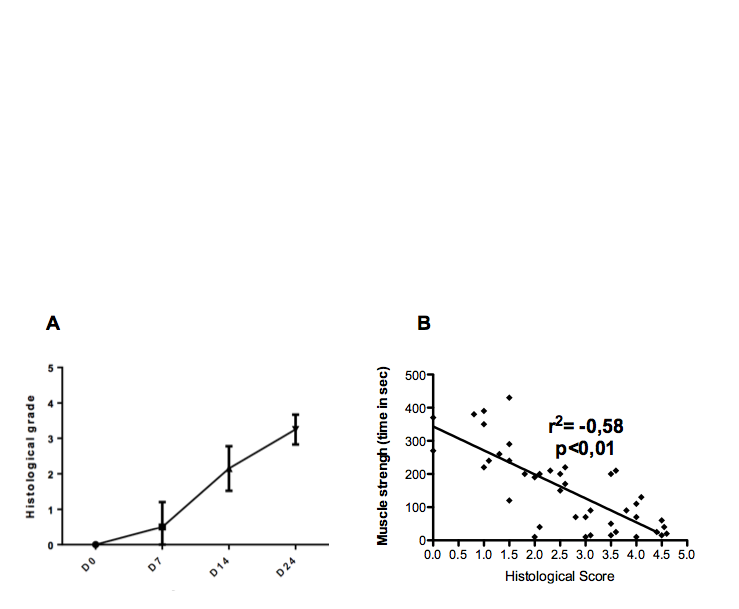

Supplement: Figure S2 — Kinetic of histological score and correlation between muscle strength (time to fall) and histological grade. A: histological score of animals over time after 3 weekly immunizations against myosin at day 0, 7, and 14 (D0, D7, and D14, respectively). B: correlation between muscle strength (time to fall) and histological grade of inflammation after curative treatment of EAM. (TIFF) [file pone.0074450.s002.tif]
